# Overview of Antimicrobial Biodegradable Polyester-Based Formulations

**DOI:** 10.3390/ijms24032945

**Published:** 2023-02-02

**Authors:** Oana Gherasim, Valentina Grumezescu, Stefan Andrei Irimiciuc

**Affiliations:** 1 National Institute for Lasers, Plasma and Radiation Physics, 077125 Magurele, Romania; 2 Academy of Romanian Scientists, Splaiul Independentei No. 54, 050044 Bucharest, Romania

**Keywords:** polylactide, poly(lactide-co-glycolide), polycaprolactone, poly(3-hydroxybutyrate-co-3-hydroxyvalerate), anti-infective therapy

## Abstract

As the clinical complications induced by microbial infections are known to have life-threatening side effects, conventional anti-infective therapy is necessary, but not sufficient to overcome these issues. Some of their limitations are connected to drug-related inefficiency or resistance and pathogen-related adaptive modifications. Therefore, there is an urgent need for advanced antimicrobials and antimicrobial devices. A challenging, yet successful route has been the development of new biostatic or biocide agents and biomaterials by considering the indisputable advantages of biopolymers. Polymers are attractive materials due to their physical and chemical properties, such as compositional and structural versatility, tunable reactivity, solubility and degradability, and mechanical and chemical tunability, together with their intrinsic biocompatibility and bioactivity, thus enabling the fabrication of effective pharmacologically active antimicrobial formulations. Besides representing protective or potentiating carriers for conventional drugs, biopolymers possess an impressive ability for conjugation or functionalization. These aspects are key for avoiding malicious side effects or providing targeted and triggered drug delivery (specific and selective cellular targeting), and generally to define their pharmacological efficacy. Moreover, biopolymers can be processed in different forms (particles, fibers, films, membranes, or scaffolds), which prove excellent candidates for modern anti-infective applications. This review contains an overview of antimicrobial polyester-based formulations, centered around the effect of the dimensionality over the properties of the material and the effect of the production route or post-processing actions.

## 1. Introduction

With the aim to overcome the current challenges of classical pharmacotherapy (drug-related pharmacological mechanisms, such as partial specificity for receptor targeting, limited control, and distribution over tissue volume, but also inappropriate or incorrect administration) [1,2,3], emerging bio-nanotechnologies enable the progress of patient-oriented and performance-enhanced therapeutic strategies. Modern pharmacotherapy relies on the synergetic association between biomedical sciences (biochemistry and biophysics, cellular and molecular biology, and physiological and pathological molecular interactions) and nanotechnology (in-depth understanding and accurate manipulation of ultra-small-scaled mater and materials), towards developing the necessary infrastructure for the implementation of personalized healthcare desideratum [4,5,6].

The progress of alternative antimicrobial therapies, which involve interdisciplinary research, implies challenging and prospective protocols that study microbial interactions [7,8,9], investigate biocidal and biostatic mechanisms [10,11,12], and develop functional anti-pathogenic treatments [13,14,15]. The use of biopolymers as unconventional antimicrobial platforms (either as intrinsic anti-pathogenic agents [16,17,18] or as active drug carriers [19,20,21]) is of great significance when considering conventional antibiotherapy efficacy control, combating the alarming occurrence of drug-resistant pathogens, and limiting or eliminating the clinical implications of biofilm-related complications.

When developing new and effective antimicrobials, polymeric platforms provide indisputable advantages regarding the manufacturing of modern pharmacologically active formulations. The main targeted aspects are the protection of embedded drugs, targeted and triggered control over localized tissue distribution, specific and selective targeting of cellular receptors, reduced or mitigated collateral/side effects, and pharmacological efficacy [22,23]. In particular, the design and fabrication of pharmacological formulations based on biodegradable polyesters is of great importance for the progress of personalized biomedicine. Their intrinsic peculiarities are enabling modern and effective strategies for molecular diagnosis and treatment [24,25], anti-infective therapy [26] and cancer management [27,28], and tissue engineering and regenerative medicine [29,30].

**Figure 1:**
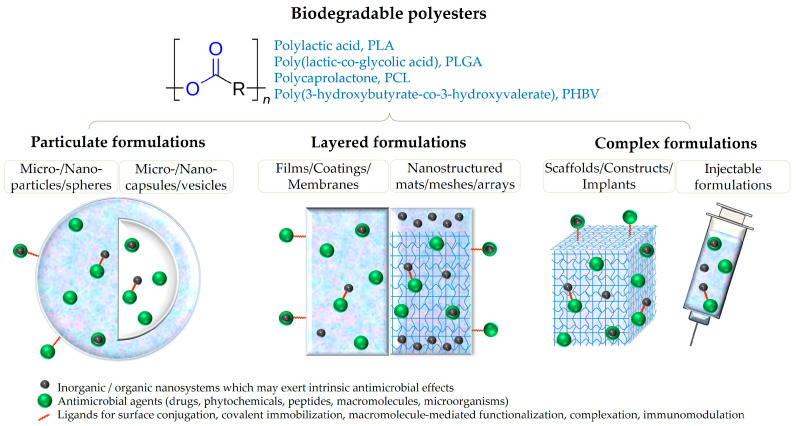
Schematic representation of antimicrobial biopolyester-based formulations.

An updated literature survey on the most recent reports in new and efficient antimicrobial formulations based on biodegradable polyesters is herein proposed. Our study considers the most explored and prolific polyester candidates for developing modern anti-infective platforms, evidencing their use as particulate, layered, or complex formulations (Figure 1).

## 2. Biopolyesters

The use of biodegradable polyesters in biomedicine relies on their versatile characteristics, including biomimicking composition and microstructure, tunable degradability and physiological metabolization, and complex functionality and biological performance [31,32].

**Figure 2:**
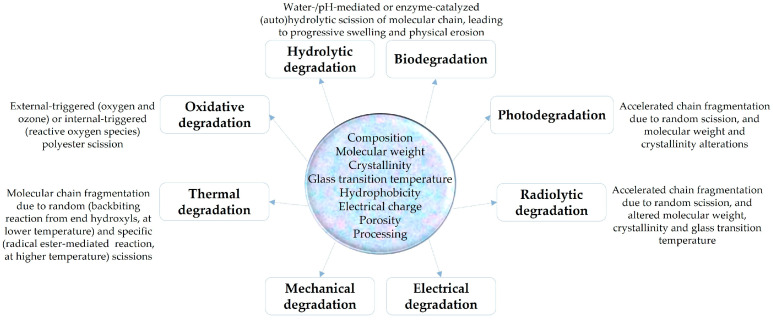
Schematic representation of degradation mechanisms in biopolyester-based formulations.

The degradation of polyesters (Figure 2) represents the essential feature of such biopolymers, conferring excellent biocompatibility of polyester-based platforms (due to the physiological processing and natural elimination of the hydrolysis-resulted degradation products) and resulting in promising pharmacologically active formulations [33,34].

The degradation of biopolyesters is mediated by the (auto)catalytic hydrolytic or enzymatic degradation of constituent ester linkages and depends on specific intrinsic physicochemical aspects, such as composition and molecular weight, hydrophobicity, crystallinity, and glass transition temperature [35,36,37]. Slower drug release and delayed polymer degradation have been evidenced in the case of biopolyesters with high molecular weight (due to low chain mobility and reduced swelling and solubility) and high hydrophobicity (due to increased hydrolytic resistance) [38,39,40]. Even with the high drug-loading efficiency reported for highly crystalline polyesters, the consequences of increased crystallinity and reduced glass transition temperature (through the drug-plasticizing effect) are seen in the reduced drug release and polymer degradation rates [41,42,43].

Polylactide (PLA) and poly(lactide-co-glycolide) copolymer (PLGA) are key representatives of polyester-based therapeutics. Their intrinsic mechanical behavior, thermoplastic properties, tunable solubility and degradation, and excellent biocompatibility enable their implementation in the fabrication of modern biomedical platforms, such as biodegradable and bioresorbable implantable devices [44,45], tailorable stimuli-responsive pharmaceuticals [46,47], and patient-oriented tissue substitutes or augmentations [48,49]. In addition, retard formulations [50] and long-term medical devices [51,52] can be developed by exploring the intrinsic features of polycaprolactone (PCL) and poly(3-hydroxybutyrate-co-3-hydroxyvalerate) copolymer (PHBV), namely, thermoplasticity- and crystallinity-related hydrophobicity and the reduced degradation rate. Moreover, the piezoelectric effect of biodegradable materials and devices based on naturally derived PLA and PHBV represents a strong argument for their incorporation in smart therapeutic platforms [53,54].

The degradation of PLGA and PHBV copolymers can be tailored through the molecular weight and ratio of constituent monomers. For instance, the fastest degradation rate of PLGA copolymers is for the 50:50 lactide–to–glycolide (LA/GA) representative, which decreases with the increasing of the lactide constituent, due to the LA-mediated abundance of hydrophobic methyl side groups and GA-mediated reduced crystallinity [55,56]. In PHBV, higher degradation rates have been evidenced for copolymers with more hydroxyvalerate (HV) content, because of the HV-mediated reduced crystallinity and accelerated hydrolysis [57,58].

Additives (drugs, biomolecules, and inorganic and organic reinforcers) impact the stability and degradability of biopolyesters at a microstructural level [59,60,61], but their degradation profile can also be modulated by external factors, such as pH [62], temperature [63,64], electromagnetic radiation [65,66], and enzymes [67,68]. 

In [69], the effect of femtosecond laser irradiation on the biodegradability of PLGA films was shown. Comparisons of the 800 nm and 400 nm heat-affected zones for irradiation have shown that infrared irradiation provides a small affected area. The results were also corelated with the degradation rate of the PLGD when 400 nm irradiated films had a considerably higher degradation rate when compared with the 800 nm ones. The behavior was explained by the decrease in molecular weight as a result of the dissociation of the chemical bonds. The aforementioned results are amongst the first reports on the dependence of the degradation rate of biodegradable polymers on the irradiation wavelength. 

## 3. Particulate Formulations

Biodegradable polyesters represent attractive candidates for the fabrication of new and efficient antimicrobials. Conventional and modified emulsification protocols are usually employed for the synthesis of biopolyester-based micro-/nano-sized particles [70,71], but superior outcomes have also been reported by using nano-precipitation [72,73] and microfluidic and membrane emulsion [74,75]. The proper synthesis method (the selection of which is strongly influenced by the stability, half-lifetime, hydrophilicity, or lipophilicity of the antimicrobial payload) [76,77] facilitates the formation of compact or porous spheres and capsules with intrinsic microstructures that are directly related to the degradation and release profiles.

The chemical reactivity and compositional versatility of such systems enable impressive possibilities for conjugation or functionalization (micro-/nano-particles), loading or entrapment (compact or porous micro-/nano-spheres), and encapsulation (micro-/nano-capsules) of the antimicrobial cargo, which may consist of drugs, phytochemicals, biomolecules, inorganic nanosystems, and macromolecules [78,79,80]. The same features open the path for additional surface modifications and coatings, with the final goal to fabricate multifunctional formulations with active targeting abilities. The surface of biopolyester-based particulate formulations can be (bio)chemically tailored to target microbial pathogens (electrostatic interactions or specific binding to surface molecules overexpressed by microbial cells) [81,82,83] or infected tissues (selective coupling to specific tissue receptors, including the stealth effect) [84,85,86]. Besides such targeting abilities, most polyester-based formulations exert their therapeutic action through passive targeting, which is mainly a concentration-dependent effect that occurs at the infection site due to the increased retention and accumulation caused by the vascular and lymphatic impairment [87,88,89].

PLA-based pharmacological platforms possess reduced immunogenicity (owing to their intrinsic degradation mechanisms, which results in non-toxic and metabolically active secondary products) and enable controlled and/or targeted delivery mechanisms [90,91]. As a result, several PLA-based formulations have been approved by regulatory institutions for safe use in clinical practice [92,93]. 

Prominent inhibitory effects on Gram-negative strains were reported by encapsulating eugenol and linalool in PLA particles [94], due to hydrophobicity-mediated interactions between particles and microbial outer membranes. By contrast, the embedding of carvacrol (53.9%) within polyethyleneimine-coated PLA nanoparticles (114.7 ± 1.02 nm) resulted in long-term antimicrobial activity against Gram-positive strains [95], through enhanced cellular uptake facilitated by the cationic-charged nanoparticles. Further, a prolonged anti-staphylococcal efficacy has been shown for gentamicin-loaded PLA microspheres [96].

In addition, in the case of bone fixing screws modified with layered coatings of PLA films and vancomycin-loaded PLA nanospheres, the long-term release of the antibiotic (up to 24 days) led to the eradication of *Staphylococcus aureus* (*S. aureus*) during the contamination phase, followed by a drastic inhibition of the staphylococcal bacterial biofilm [97].

Micro-/nano-sized PLGA-based platforms have an essential role in emerging modern pharmacotherapy, with several clinically approved formulations [98,99]. The anti-infective clinical potential of PLGA systems, evidenced by efficient drug entrapment and effective drug release, has been evidenced for various antibiotics, including ceftiofur [100], doxycycline [101,102], gentamycin [103], rapamycin [104], rifapentine [105,106], and vancomycin [107].

The efficient loading of tobramycin/dioctyl sulfosuccinate conjugates within PLGA nanoparticles (>89%) determined significant bactericidal effects against *Pseudomonas aeruginosa* (*P. aeruginosa*), even when using sub-inhibitory antibiotic concentrations [108]. Complementary studies evidenced that the antimicrobial efficacy of ciprofloxacin, florfenicol, and cefpodoxime proxetil was enhanced following their incorporation into particles of PLGA conjugated with polysorbate surfactant [109], Eudragit (enteric methacrylic polymer) [110], and (CS) chitosan/Eudragit [111], respectively.

While exhibiting important antibacterial and hemostatic effects, the sustained release (up to 2 weeks) of tylotoin peptide molecules from CS-coated PLGA nanocapsules determined reduced inflammatory events and accelerated healing in full-thickness skin wounds [112]. PLGA nanospheres were recently proposed to overcome solubility limitations of plant-derived gentiopicroside, demonstrating important anti-staphylococcal effects and accelerated healing rates in diabetic wounds [113]. Considerable bactericidal and bacteriostatic effects have also been reported by encapsulating cinnamaldehyde (33.20 ± 0.85%) in PLGA/CS nanoparticles [114], while porous PLGA microspheres embedding antimicrobial peptides showed promising potential for the local management of bone infections [115].

Composite PLGA/CS microspheres provided a prolonged release of antimicrobial peptides and determined subsequent long-lasting antibacterial effects (almost 3 months) against microorganisms from the oral flora [116]. Aiming to reduce the cariogenic risk, effective anti-streptococcal platforms have been developed by incorporating chlorhexidine salts into PLGA microparticles [117] and PLGA-coated mesoporous silica nanoparticles [118]. The prophylactic potential of PLGA nanoparticles modified with antimicrobial peptides on periodontal disease has also been highlighted; such platforms prevented the adhesion of oral microorganisms to the endogenous streptococcal community and, consequently, inhibited the formation and development of polymicrobial biofilms [119].

Bacteriophage-loaded PLGA microparticles exhibited pronounced bactericidal effects against planktonic and sessile *P. aeruginosa*. Following their murine inhalation, the as-developed systems induced a drastic reduction of the pulmonary microbial community and an effective control over pneumonia-associated pulmonary and hepatic complications [120]. The intracellular release of clarithromycin [121] and amikacin-moxifloxacin complex [122] from PLGA nanocapsules has shown promising potential to combat opportunistic infections associated with lung disease. Inhalable platforms based on curcumin-loaded PLGA nanoparticles embedded in drug matrix (tobramycin/ciprofloxacin/azithromycin and N-acetylcysteine) have been proposed for the multivalent treatment of lung infections, as evidenced by their cumulative anti-inflammatory, antibacterial, and mucolytic effects [123].

The triggered release of clarithromycin from hybrid microparticles based on the magnesium core, antibiotic-loaded PLGA layer, and CS coating significantly reduced the gastric level of Helicobacter pylori [124]. With a similar goal, the targeted therapeutic potential of amoxicillin-loaded nanocapsules based on PLGA and CS derivatives has been reported [125]. PLGA nanocapsules loaded with meropenem–cyclodextrin complex provided antibiotic stability under acidic conditions and enabled controlled antibiotic release under neutral conditions. As a result, they have been proposed as efficient anti-infective platforms for the gastrointestinal tract [126]. Considerable anti-amoebic effects have been reported for the efficient encapsulation of gallic acid (82.86%) into PLGA particles (~100 nm) [127].

Though it is more difficult to safely assess their efficiency, promising antiviral outcomes of biopolyester-based formulations have also been reported (Table 1).

Highly stable PCL-based micelles showed efficient loading of luteolin (97.3% ± 1.1%) and ofloxacin (64.23%) and determined their prolonged release (up to 8 hours), being proposed as tablet formulations for the local treatment of fungal infections [143] or as particulate systems for treating ocular infections [144], respectively. Sustained and pH-responsive drug release was evidenced for negatively charged PCL nanoparticles encapsulating cefotaxime, determining important anti-fouling activity against bacterial pathogens [145]. 

The surface coating of urinary catheters with chlorhexidine-loaded PCL nanospheres (152 ± 37 nm) [146] and PEG-block-PCL micelles (40.21 ± 3.85 nm) [147] proved an effective and prolonged strategy for reducing the contamination by and colonization of uropathogenic microorganisms. Complementary studies evidenced that nanosystems-based multilayer coatings determined long-term antibacterial and anti-biofilm effects through the sustained release of chlorhexidine (for up to 4 weeks), while exhibiting good biocompatibility and reducing the longevity-related limitations of catheterization (encrustation and crystal deposition) [26,148].

The particular degradation kinetics of PHBV-based biomaterials are beneficial for the successful development of therapeutically effective formulations, with promising outcomes for tissue engineering [52,149] and modern pharmacotherapy [150,151].

Impressive therapeutic efficacy and preventive action have been reported in *Salmonella Typhimurium* systemic infection following the intramuscular administration of ceftiofur-loaded PHBV microparticles. The pharmacokinetic and toxicological studies revealed no changes in the biochemical and hematological parameters, and a lack of hepatotoxic and nephrotoxic effects, respectively [152]. Nano-magnetite-loaded PHBV/ceftiofur composite nanoparticles significantly inhibited the development of *Escherichia coli* (*E. coli*), while exhibiting high cytocompatibility in human hepatocytes. The as-developed hybrid nanosystems (243.0 ± 17 nm) have been evaluated as multifunctional platforms for the local management of infections, by means of magnetic resonance imaging, magnetic hyperthermia, and controlled release of the antibiotic [153]. The multi-faceted functionality of PHBV–Fe_3_O_4_ (magnetite) composites have also been reported in the case of biopolymer microspheres loaded with magnetic nanoparticles functionalized with lauric and oleic acids [154,155].

The encapsulation of epirubicin within composite PHBV–PEG (polyethylene glycol) particles determined important antibacterial effects against Gram-positive and Gram-negative strains, with superior efficiency to equivalent concentrations of free drug. The obtained nanosystems (152.3 ± 0.6 nm) exhibited fast and sustained pH-dependent drug release, as evidenced under acidic and neutral physiologically simulated conditions (2 and 8 days, respectively) [156]. Highly cytocompatible PHBV–CS spheres proved to have potentiating effects on different bioproduced antibiotics (against various clinically relevant bacterial strains); however, only kanamycin-loaded composites exhibited reduced pro-inflammatory effects beneficial for the modulation of the healing process and microbicidal mechanisms. 

Owing to their superior mechanical properties and tunable degradability, PHBV-based formulations are extensively investigated regarding the development of biomaterials and devices for restorative and regenerative applications of bone tissue. With the aim to reduce the bacterial contamination and colonization susceptibility of metallic implants, levofloxacin-loaded PHBV microspheres were embedded within alginate matrix and validated as compact coatings that exert sustained bactericidal effects against *E. coli* [157]. Superior antimicrobial efficacy and prolonged release of cinnamaldehyde (7 days) and vancomycin (4 days) have been reported in the case of PHBV-based microspheres embedded with mesoporous vitroceramic nanoparticles [158] or loaded within vitroceramic scaffolds [159], respectively.

The effects of WS_2_ nanotubes addition of the mechanical properties of biodegradable polymers (PLLA) were also investigated [160]. The best improvement of the mechanical properties are shown for INT–WS_2_ addition up to 0.5 wt. %. The addition of nanotubes in the composition of polymers reportedly also reduces the friction coefficient of the polymer–nanotube composite. 

Additionally, no hindering of viscosity or polymer matrix moduli is reported based on rheological sties performed on the composites. The polymers’ bond stretching was highlighted by the use of Raman Spectroscopy, with no observable interference with respect to the polymerization process by the insertion of WS_2_ nanotubes. Increases in the PLLA’s crystallinity are also reported by use of differential scanning calorimetry investigations and confirmed by X-ray diffraction with nanotubes acting as nucleation centers, thus transforming the composite into a semi-crystalline material.

More than extending the safe use of conventional antimicrobials and limiting their negative side effects, biopolyester-based particle formulations represent ideal candidates for modern anti-infective therapy, contributing to the emerging clinical evaluation of more effective, comfortable, and compliant treatments. Intrinsic and circumstantial biodegradability, but also excellent biocompatibility and thermoplasticity, represent key aspects that highlight their promising use for the development of modern and efficient antimicrobial platforms, susceptible to various administration routes. Being biosafe and biodegradable materials, polyesters have a great potential for the commercial fabrication of particulate antimicrobials, providing specific, selective, controlled, targeted, and personalized anti-infective effects.

## 4. Layered Formulations

Fabricating protective and highly biocompatible surface-modifying coatings [161] and nano-sized/-structured bidimensional formulations [162,163] is an attractive and emerging strategy to modulate the microbial susceptibility of commercial medical devices and develop new anti-infective devices, respectively. Given their tunable biomechanics, thermophysics, and biochemistry, but also their versatile processability, polyesters are indisputable candidates for the fabrication of such active carriers or enhancers for local antimicrobial effects.

**Figure 3:**
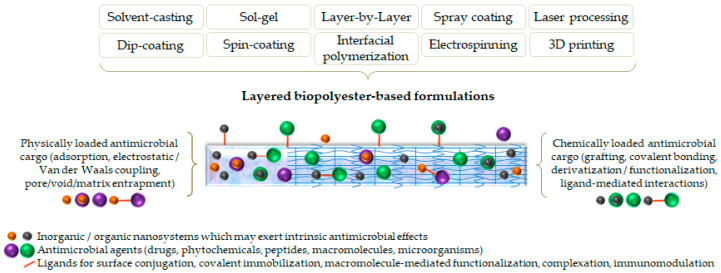
Schematic representation of synthesis methods used to fabricate biopolyester-based layered formulations.

Various synthesis methods have been employed to obtain antimicrobial coatings for biomedical materials and devices [164,165] (Figure 3). The degradation and release profiles of such formulations can be tuned at a microstructural and morphological level, depending on the therapeutic effect and final use. Furthermore, boosted bioactivity and additional functionality may be achieved by means of polyester coatings.

PLA films were reported to have the role of active matrices for the release of metallic (silver) and oxide (zinc and titanium oxide) nanoparticles with intrinsic anti-pathogenic activity, exhibiting pronounced inhibitory effects against the *E. coli* strain [166]. By contrast, increased anti-staphylococcal efficacy was obtained in the case of layered films of PLA and thymol-encapsulated zein-chitosan solid particles [167]. Enhanced anti-biofilm activity against *S. aureus* was also demonstrated for PLA films embedded with stearate-stabilized silver nanoparticles [168] and nano-magnetite conjugated with eucalyptus essential oil [169], while exhibiting excellent biocompatibility with respect to human-derived endothelial cells and mesenchymal stem cells, respectively. 

Excellent bioactivity and prolonged anti-staphylococcal efficacy have been reported for hybrid structures based on PLA films reinforced with gentamicin-oaded coralline hydroxyapatite (HAp) nanoparticles (as evidenced up to 4 weeks [170,171]) and PLA–PVA (polyvinyl alcohol) microsphere coatings entrapping usnic acid [172]. 

The immobilization of recombinant antimicrobial peptides in PLA membranes has been successfully evaluated for topical bactericidal use, with additional beneficial effects on the adhesion and proliferation of human fibroblasts [173]. Concerning the fabrication of antimicrobial wound dressings, PLA/gelatin nanofiber mats demonstrated a sustained release of phyto-conjugated silver nanoparticles, but also proper mechanical and gelation properties [174]. Antibacterial and antioxidant activity have been also reported for PLA–PEG composite films incorporating silver nanoparticles conjugated with phytochemicals [175], while PLA/PEG nanofibrous mats were recently proposed for the transdermal administration of acyclovir against HSV type-1 infection [176]. Non-toxic nanocomposites with ultraviolet-light barrier properties have been developed by the impregnation of cinnamaldehyde within PLA/lignin nanoparticle films [177].

An increased antimicrobial efficiency has also been reported in the case of biodegradable PLGA membranes loaded with phytochemicals, such as cinnamaldehyde and carvacrol [178], aloe vera [179], and thymol [180]. The immobilization of eugenol and clove essential oil within PLGA films was beneficial for potentiated anti-biofilm activity against enterohemorrhagic *E. coli* [181]. Excellent efficiency against mature microbial biofilms has been evidenced in the case of nanostructured coatings based on PLGA microspheres or PLGA films embedded with phytochemical-conjugated [182] and antibiotic-functionalized magnetite nanoparticles [183,184]. Superior anti-staphylococcal efficacy was also reported for hybrid coatings of PLGA–PVA microspheres loaded with usnic acid and nano-magnetite [185].

Using graphene oxide filler proved effective for increasing the hydrophilicity and modulating the adsorption ability of biomolecules (antimicrobial peptides and growth factors) in PLGA films. Such nanostructured formulations showed important antibacterial effects against opportunistic strains and accelerated healing and tissue regeneration ability, being evaluated as promising platforms for the infection-free regeneration of wounds [186]. Superior mechanical properties and enhanced acyclovir loading efficiency were reported for PLGA/PCL nanofibrous mats, in comparison with bare PLGA materials. Besides exhibiting short-term viral inhibition, the reduced polymer degradation and continuous drug release resulted in long-term protection against viral transmission of HSV type-2 infection [187].

HAp/PLGA coatings entrapping ceftriaxone and cefuroxime are suitable materials for the normal development of osteoblasts [188] and showed enhanced anti-biofilm activity against the *E. coli* strain. Bacterial inhibition has also been demonstrated in the case of multi-layered membranes consisting of either collagen nanofibers loaded with PLGA nanoparticles and aspirin or curcumin-functionalized collagen nanofibers. Besides their antibacterial efficiency, the as-developed hybrid structures exhibited important osteogenic activity, being proposed for reducing infection-associated complications in guided bone regeneration [189]. Hybrid films consisting of vancomycin-loaded chitosan hydrogel and PLGA nanoparticles loaded with osteoinductive protein enabled the sequential release of biosubstances (2 and 12 days, respectively) and resulted in accelerated and uncomplicated osteointegration [190]. Therapeutic effects (stable prosthetic fixation and faster healing) and prophylactic effects (effective control of methicillin-resistant *S. aureus* contamination for up to 28 days) were revealed by the encapsulation of linezolid within PLGA nanofibrous membranes [191].

PCL films loaded with ciclopirox olamine-modified vermiculite determined the long-term inhibition of bacterial and fungal biofilms, while the addition of zinc oxide (ZnO) nanoparticles resulted in potentiated anti-biofilm effects [192]. Highly hemocompatible PCL films embedded with copper oxide nanoparticles have been evaluated as promising candidates for the management of diabetic foot ulcers infected with opportunistic methicillin-resistant *S. aureus* [193].

PCL/poly(ethylene succinate) polyester mixture provided extended release of the biocide agent and prolonged antibacterial effects when used as a shell coating for drug-loaded polyvinylpyrrolidone core nanofibers, leading to the development of bacteria-degradable nanofibrous membranes for wound care management [194]. As hydrophilicity and water retention are important aspects when designing wound dressings, the addition of gelatin proved beneficial for increasing these parameters in PCL nanofibers. Meshes based on ciprofloxacin-loaded PCL core/tetracycline-loaded gelatin shell fibers showed improved mechanical properties (when compared to uniaxial membranes) and exhibited long-lasting antibacterial effects [195]. Also aiming at the development of efficient topical platforms, biocompatible PCL nanofiber arrays decorated with ZnO tetrapod nanoparticles demonstrated important and sustained dose-dependent antiviral effects against both type-1 and type-2 HSV [196]. The successful revaluation of anti-pathogenic phytochemicals in the development of antimicrobial alternatives has been demonstrated in the case of PHBV films loaded with rosemary and green tea extracts [197], oregano essential oil [198,199], and eugenol-encapsulated mesoporous silica nanoparticles [200].

Nanostructured coatings of PHBV–PVA microspheres entrapping eugenol-functionalized nano-magnetite exhibited important anti-adherence and sustained anti-biofilm effects (evidenced against *S. aureus* and *P. aeruginosa*), showing superior cytocompatibility with respect to human endothelial cells [201]. Prolonged anti-biofilm efficiency against Gram-positive and Gram-negative pathogens, as well as excellent biological behavior on osteoblasts and endothelial cells, was reported for coatings based on lysozyme-embedded PHBV/PEG microspeheres [202].

With the aim to fabricate performance-enhanced topical formulations, PHBV nanofibrous membranes were reinforced with hybrid systems consisting of cellulose nanocrystals (NCC) and ZnO, which resulted in improved mechanical strength and thermal stability, and antibacterial efficiency (demonstrated against both *E. coli* and *S. aureus* strains) [203]. In a similar study, the cumulative release of tetracycline (~80%) from PHBV and NCC composite membranes grafted with methacrylic polymer was attained after 4 days of testing under physiologically simulated conditions. Under weakly acidic conditions or by increasing the temperature, the antibiotic release period was reduced to only 2 hours [204]. The reinforcement of PHBV/alginate films with graphene nanoplatelets led to the formation of thermally stable, highly hydrophobic, and electrically conductive biomaterials, which exhibited important action against a bacteriophage-based model of enveloped viruses [205].

Volova et al. [206] investigated the effect of CO_2_ laser irradiation of PHA’s films produced by the solvent cast technique. Two different working modes were considered—a continuous wave using a power of 3 W and a scanning speed of 2 m/s and a pulsed wave using a power of 13.5 W and a scanning speed of 1 m/s—for the irradiation of poly-3-hydroxybutyrate in a mixture with 30% 4-hydroxybutyrate, 3-hydroxyvalerate, or 3-hydroxyhexanoate. The polyhydroxyalkanoates (PHA) films offer a wide range of thermal, mechanical, or molecular properties, with the irradiation affecting all of their key parameters on top of biocompatibility. For example, the poly-3-hydroxybutyrate (P(3HB)) films present a decrease in contact angle from 92 to 80, while both the surface energy and the roughness increase from 30 to 57 mN/m and from 144 up to a maximum value of 290 nm, respectively. All PHA’s films irradiated in continuous mode present a decrease in contact angle down to 80 and an overall roughness increase up to 45 mN/m. The pulsed irradiation regime defines stronger morphological changes, as expected due to the higher local beam power, and thus a steeper decrease in contact angle down to 67. Assessing the cell metabolic activity for a culture of mouse fibroblast proved the advantage of pulsed treatment, which increased the number of viable cells with a factor of 1.5. These new results offer the perspective of targeted surface modification for cell attachment control.

## 5. Complex Formulations

The successful use of biopolyesters in fabricating more complex constructs (scaffolds, sponges, and foams) and topical formulations (gels) has also been evidenced [207,208]. 

Owing to their facile processability and impressive clinical outcomes, biodegradable polyesters have been extensively used for fabricating three-dimensional constructs for implantable devices and regenerative medicine. More than representing adequate mechanical and biochemical support for beneficial interactions with physiological biomolecules and resident cells, such constructs own the indisputable structural advantage. The porous microstructure of scaffold-type formulations facilitates local nutrient transport and provides adequate biomimetic support for cellular ingrowth, but also promotes local vascularization and tissue repair/regeneration.

Highly biocompatible and bioactive systems able to recover the integrity and functionality of damaged tissues through their restoration, replacement, or regeneration can be fabricated by properly adjusting the composition, microstructure, wettability, surface charge, morphology, topography, and reactivity of biopolyester-based platforms [209,210]. In addition, their advanced biofunctionality can be modulated by using composite constructs of polyesters and natural polymers, such as polysaccharides and proteins [211,212].

Excellent bioactivity and prolonged anti-staphylococcal efficacy have been reported for hybrid structures based on collagen, nano-Hap, and vancomycin-loaded PLA scaffolds (18 days) [213]. Highly biocompatible architectures based on PLA, barium sulphate particles embedded in polydopamine, and levofloxacin with excellent inhibitory activity against the development of *S. aureus* have been proposed as bone fixation devices [214].

The incorporation of green indocyanine into PLA nanofibrous networks has been attempted in order to eradicate bacterial contamination and colonization of chronic wounds by means of photodynamic therapy. The newly developed materials are exhibiting accelerated degradation under alkaline conditions or in the presence of proteases, promoting physiological cellular events and encouraging pro-angiogenic effects [215].

Electrospun PLGA scaffolds modified with growth factor and antimicrobial peptide have been proposed for the accelerated healing of cutaneous wounds, while reducing the risk for opportunistic contamination with *E. coli* and *S. aureus* [216]. Considerable antimicrobial effects have been reported by the immobilization of ciprofloxacin within PLGA/alginate nanofibrous networks [217] and PLGA nanoparticles embedded into PVA hydrogels [218]. Moreover, demineralized bone matrix loaded with PLGA microparticles co-encapsulating vancomycin and HAp nanoparticles was proposed as osteogenic and highly efficient antibacterial fillers for infected bone defects [219].

For altering the hydrophobic nature of polyester, PCL/gelatin [220,221,222] and PCL/chitosan [223] composite scaffolds, with suitable mechanical behavior for skin tissue use, were developed. The porosity-related features (release profile, swelling, and permeability) of such constructs were beneficial for infection-free wound-healing applications when loaded with antibiotics and phytochemicals. Similar outcomes were also evidenced for juglone-modified PCL scaffolds [224]. Improved biomechanics and hydrophilicity were reported for PCL/gelatin scaffolds reinforced with calcium phosphate-modified graphene oxide. Exhibiting important antibacterial activity, the clindamycin-loaded osteoinductive scaffolds represent promising candidates as electrically actuated bone substitutes [225].

The release of vancomycin over a period of 22 days was evidenced in the case of composite structures based on PHBV, nano-diamond, and nano-HAp [226]. Recently, bilayer PHBV/pullulan nanofibrous scaffolds were developed as bacteria-repellent formulations for wound-healing applications. While the polyester layer provided suitable microstructure for increased water and oxygen retention and suitable architecture for cellular proliferation and migration, the hydrophilic polysaccharide layer acted as a protective membrane against bacterial transmission [227]. Enhanced and sustained antibacterial activity has been evidenced by cephalexin-loaded PHBV nanofibrous sheets against different methicillin-resistant *S. aureus* strains, both in cellular and animal models. The as-developed dressing material has been evaluated as a biosafe platform for the efficient treatment of opportunistic infections in chronic diabetic foot ulcers [228].

Besides representing active carriers or enhancers for local antimicrobial treatment, The biomechanical compliance, biomimetic microstructure, and bioresorbable ability of such formulations represent key aspects promoting biodegradable polyester in designing and fabricating functional tissue substitutes.

Though exhibiting optimal biomechanical properties and biodegradability, the hydrophobicity, slow degradation rate, and drug release profiles of biopolyester-based formulations should be properly tuned for specific biomedical uses. In this regard, their modification with highly hydrophilic and stimuli-responsive polymers represents an attractive strategy to fabricate advanced platforms, as confirmed by several in vivo and ex vivo studies (Table 2).

Magnetic nanoparticles have been at the center of an impressive number of studies, as they have been categorized as reliable candidates for a wide range of applications in biomedicine. Their attractive intrinsic physical properties, often in combination with desired high biocompatibility and low immunogenicity, have the potential to tackle modern issues in nanotechnology and materials engineering directed towards biomedicine. Recent reports [240,241,242] have presented different routes to improve and highlight the properties of these magnetic nanoparticles, either by thin film coatings, sensors, or drug carriers or as components in polymer structures. Various applications have been developed based on their unique properties in order to induce spatial displacement on nanometer scales for cell seeding, materials engineering, or targeted drug delivery [243]. While there are reports [244,245,246,247] that present control over the functionality of certain cells by using magnetic nanoparticles, limitations have risen from adverse effects on the cells, which are yet to be understood [248]. Recent reports [249,250,251] on magnetic actuation have shown that there are promising results towards controlling mesenchymal stromal cells or other cells aimed at bone or cardiovascular issues, for example, bone tissue engineering performed by means of magnetic nanocomposite (nanoparticle-embedded polymers) engineering via thin films, scaffolds, or implants [252]. The main goal of these studies is to obtain differential selectivity for specific molecules towards cell functionalization control under magnetic field action [253,254,255]. 

Simultaneously, a continuous effort has been performed towards regenerative medicine by controlling the healing and repair process for certain biological structures by phenotypic modulation of stromal cells via matrix tailoring of the physical properties or by molecular targeting of intercellular paths [256,257,258,259,260,261,262]. The development of materials with smart dynamics having the ability to aid regenerative processes at a cellular level is also done by external reactive processes, such as optical or thermoelectric stimulation, or by self-regeneration and enzymatic sensitivity control [263,264,265,266,267]. While the literature concerning tissue healing by magnetic field manipulation at a cellular level is generous, the results mostly relate to specific conditions and are very often experiment dependent in terms of the type of cell used or the stimulation of the cell with matrices with magnetic response [268,269,270]. The advantages of using magnetic nanocomposite for cell modulation have been the main driving forces behind state-of-the-art research concerning innovating and developing new strategies for tissue repair. Most utilized routes involve the control of cell behavior by functionalization, surface modulation, and cell-environment tailoring. In [271], the manufacturing of hydrogels responsive to anisotropic magnetic field tissue engineering is reported, indicating a strong correlation between the physical properties of the polymer matrix and those of the applied magnetic field. Further development of nanoparticle–polymer composites was focused mainly on increasing the response and sensitivity to the magnetic field, together with lowering the chances of biological tissue poisoning by controlling the number of magnetic nanoparticles and the cytotoxicity. 

The production of spherical Pd nanoparticles generated by pulsed laser ablation in liquid (PLAL) is reported in [272]. Different routes for NP control in size and crystallinity are attempted by utilizing different solvents, as well as different wavelengths for irradiation. Multivariable control over NP production led to the generation of NP with an average dimension of 6 nm. When the antimicrobial activity was investigated, it was reported that NP produced with 1024 nm and in methanol had a better response to Staphylococcus aureus. The antimicrobial activity is understood as Pd ions release from the NP coating, with no reported harmful effects to the cells. Besides the elevated antimicrobial activity, the encouraging report of cytocompatibility shown by estimating the bactericidal factor promotes further biological testing for these promising nanostructures. 

## 6. Conclusions

To overcome the clinical complications induced by microbial infections, known to have life-threatening side effects, conventional anti-infective therapy is generally preferred. Yet, one should note its important shortcomings concerning drug-related inefficiency or resistance and pathogen-related adaptive modifications. In this respect, advanced antimicrobials and antimicrobial devices are urgently needed.

Besides their role as protective or potentiating carriers for conventional drugs, biopolymers are characterized by an impressive ability for conjugation or functionalization, which proves beneficial to avoid collateral and side effects, and to provide targeted and triggered drug delivery, specific and selective cellular targeting, and pharmacological efficacy. It should be mentioned that biopolymers can be fabricated in various forms, i.e., particles, fibers, thin films, membranes, or scaffolds, which are demonstrated to be excellent candidates for modern anti-infective applications.

This is a comprehensive study that gathers the recent antimicrobial, polyester-based formulations, centered around the effect of the dimensionality, production route, or post processing actions on the properties of the investigated materials.

## Figures and Tables

**Table 1:** Particulate biocompatible polyester-based antiviral formulations.

System Description	Therapeutic Potential	Refs.
PLA oligomers	Inhibitory and virucidal effects against human Influenza A virus (IAV) and severe acute respiratory syndrome (SARS) virus	[128]
PLA/Ag nanocomposites (green tea extract reduced metallic particles)	Virucidal effects against human IAV and adenovirus serotype 2	[129]
PLA nanoparticles entrapping bacterial antigen adjuvanted with aluminum hydroxide	Long-lasting and efficient protection against infection caused by SARS coronavirus 2 (SARS-CoV-2)	[130]
Curcumin-loaded PLGA nanoparticles	Controlled and targeted local management of SARS-CoV-2 infection through antimicrobial photodynamic therapy	[131]
Oseltamivir-loaded PEGylated PLGA nanoparticles	Effective targeted treatment of pulmonary cancer and IAV infection	[132]
Peptide-conjugated maleimine-functionalized PLGA nanoparticles encapsulating lamivudine	Controlled and targeted local treatment of hepatitis B virus (HBV) infection	[133]
Adefovir-loaded PLGA microspheres		[134]
PLGA-CS microparticles encapsulating interferon-alpha	Effective protection against mengovirus infection	[135]
PLGA nanoparticles entrapping protein or peptide antigens and immunostimulatory adjuvants	Effective protection and targeted local treatment of IAV infection	[136]
PLGA nanoparticles entrapping viral antigen adjuvanted with pattern-recognition receptor agonists		[137]
PLGA microparticles loaded with viral nucleoprotein adjuvanted with immunostimulatory agonists and carbomer–lecithin nanoemulsion	Effective protection and targeted local treatment of IAV and SARS-CoV-2 infection	[138]
Nanoparticles of PLGA-PEG and PCL grafted with membrane receptor ligands and loaded with remdesivir	Efficient and targeted local treatment of SARS-CoV-2 infection	[139]
Blank PCL nanocapsules with Eudragit surface coating	Selective inhibitory effects against Herpes simplex virus (HSV) type-1	[140]
Cidofovir-loaded PEG-PCL nanoparticles ink formulation	Controlled and prolonged efficiency for the local treatment of human papilloma virus (HPV) infection	[141,142]

**Table 2:** Biopolyester-based gel formulations with antimicrobial activity.

System Description	Therapeutic Effects	Refs.
Ketoconazole-entrapped PLGA nanoparticles loaded into alginate-chitosan in situ gel formulations	Augmented drug permeation and sustained drug release Treatment of *Candida albicans* fungal keratitis and endophthalmitis	[229]
Norfloxacin-loaded PLGA nanoparticles incorporated within hydroxypropyl methylcellulose hydrogels	Prolonged drug release and superior biosafety profile Treatment of *P. aeruginosa* keratitis	[230]
Besifloxacin-loaded PCL/PEG nanofibrous inserts entrapped within thiolated sodium alginate		[231]
Vancomycin-loaded PLGA/Eudragit and PCL/Eudragit nanoparticles incorporated in Carbopol-based hydrogels	Prolonged drug release and superior biosafety profile Treatment of *S. aureus* keratitis	[232]
Levofloxacin-encapsulated PLGA nanoparticles embedded in prednisolone-containing CS/gelatin hydrogels	Extended dual-drug release and improved ocular bioavailability Treatment of *S. aureus* keratitis, management of endophthalmitis	[233]
Ciprofloxacin-encapsulated PLGA microspheres loaded within poloxamer/hyaluronic acid hydrogels entrapping ginsenoside	Sequential release ability (short-term release of the immunomodulatory ginsenoside and long-term release of the antibiotic) Treatment of skin infections through synergistic efficiency against methicillin-sensitive and methicillin-resistant *S. aureus*	[234]
Mupirocin-/ketoprofen-co-encapsulated mesoporous PHBV microparticles embedded in κ-carrageenan/locust bean gum hydrogels	Thermo-sensitive and prolonged dual-drug release Potential wound-healing applications	[235]
Rifampicin-loaded PHBV microparticles embedded in streptomycin-containing gellan gum hydrogels	Sustained dual-drug release Treatment of skin ulcers caused by *Mycobacterium ulcerans* infection	[236]
Vancomycin-loaded oligochitosan nanoparticles mixed with PLGA-PEG-PLGA gels	Thermo-sensitive and sustained drug release, osteogenic differentiation ability, and important antibacterial and anti-biofilm effects against *S. aureus* and *S. aureus mutans*, respectively Treatment of osteomyelitis and regeneration of infected bone tissue	[237]
Vancomycin-embedded and HAp-loaded PLGA-PEG-PLGA gels		[238]
Cefazoline-loaded PCL scaffold encapsulated in rifampicin-containing alginate hydrogels	Prolonged dual-drug release and important antibacterial and anti-biofilm effects against *S. aureus* Treatment of osteomyelitis	[239]
Osteogenic protein-entrapped PLGA microspheres loaded in vancomycin-containing CS hydrogels	Sequential release ability (fast release of the antibiotic for 2 days and sustained long-term release of the protein for 12 days) Important antibacterial activity and reduced infection-mediated inflammation caused by *S. aureus mutans*, osteogenic differentiation, and bone regeneration ability	[191]

## Data Availability

Not applicable.
